# SLAM and 3D Semantic Reconstruction Based on the Fusion of Lidar and Monocular Vision

**DOI:** 10.3390/s23031502

**Published:** 2023-01-29

**Authors:** Lu Lou, Yitian Li, Qi Zhang, Hanbing Wei

**Affiliations:** 1 School of Information Science and Engineering, Chongqing Jiaotong University, Chongqing 400074, China; 2 Guangdong Haoxing Technology Co., Ltd, Foshan 528300, China; 3 School of Mechatronics and Vehicle Engineering, Chongqing Jiaotong University, Chongqing 400074, China

**Keywords:** SLAM (simultaneous localization and mapping), multi-sensor fusion, monocular vision, Lidar, 3D reconstruction

## Abstract

Monocular camera and Lidar are the two most commonly used sensors in unmanned vehicles. Combining the advantages of the two is the current research focus of SLAM and semantic analysis. In this paper, we propose an improved SLAM and semantic reconstruction method based on the fusion of Lidar and monocular vision. We fuse the semantic image with the low-resolution 3D Lidar point clouds and generate dense semantic depth maps. Through visual odometry, ORB feature points with depth information are selected to improve positioning accuracy. Our method uses parallel threads to aggregate 3D semantic point clouds while positioning the unmanned vehicle. Experiments are conducted on the public CityScapes and KITTI Visual Odometry datasets, and the results show that compared with the ORB-SLAM2 and DynaSLAM, our positioning error is approximately reduced by 87%; compared with the DEMO and DVL-SLAM, our positioning accuracy improves in most sequences. Our 3D reconstruction quality is better than DynSLAM and contains semantic information. The proposed method has engineering application value in the unmanned vehicles field.

## 1. Introduction

SLAM (simultaneous localization and mapping) technology mainly uses sensors such as cameras, Lidar, IMU, and GPS to locate mobile robots in an unknown environment and maps simultaneously. SLAM can be classified into visual SLAM and Lidar SLAM, depending on the external sensor.

Generally, cameras are used in visual SLAM to obtain rich visual information, which performs great advantages and potential in object detection, recognition, and environmental semantic understanding [1]. However, the quality of the images collected by the cameras is greatly affected by the light, therefore, visual SLAM has low positioning accuracy in scenes with poor illumination. On the other hand, the object information collected by Lidar is represented as a series of scattered point clouds with accurate angle and distance information. Because of the depth information contained in Lidar scans, Lidar-based SLAM helps mobile robots perform tasks such as path planning and navigation. However, unstructured Lidar points cannot present scene textures; low-texture environments such as long corridors will make trouble for Lidar SLAM [2]. As they appear to be complementary, and their fusion will be able to balance their respective major weaknesses. Using both visual and Lidar sensors can reduce local uncertainty and cumulative drift [3].

In the process of 3D reconstruction, the workload of extracting semantics and attributes from data is the largest [4]. Considering the rapidly changing environment of outdoor driving, manually annotated semantic maps may cause vehicles to perform insufficient environment recognition operations, resulting in unsafe situations. Automated 3D semantic reconstruction could reduce labor costs and improve driving safety. To improve the positioning accuracy and quality of reconstructed maps, the fusion of semantic images and Lidar should be considered.

In this paper, we propose a SLAM and 3D semantic reconstruction method based on the fusion of Lidar and monocular vision, which fuses monocular images and Lidar point clouds conveniently and efficiently for outdoor environments. We combine image features and accurate depth information to achieve robust and high-precision positioning of unmanned vehicles, combining semantic images and Lidar point clouds to reconstruct high-quality 3D maps of large outdoor scenes in a convenient and intuitive method. The contributions are as follows:

(1) We propose a projection and interpolation method to fuse low-density Lidar point clouds with semantically segmented images, and semanticize the point clouds corresponding to the semantic images.
(2) We propose a SLAM method based on the fusion of Lidar and monocular vision, which uses the upsampled point cloud to provide depth information for image feature points and improve localization accuracy.
(3) To solve the sparsity problem of mapping, we propose a 3D dense reconstruction method, which uses fused data to reconstruct dense semantic maps of the outdoor environment while localizing.

## 2. Related Work

### 2.1. Single-Sensor SLAM

Visual SLAM mainly uses cameras as external sensors to obtain a rich texture, color, shape, and other environmental information. ORB-SLAM [5] is a monocular vision SLAM based on ORB feature points. The whole system includes three parallel threads of tracking, local mapping, and closed-loop detection. Through epipolar constraints, triangulation, nonlinear optimization, and based on bag of words, it enables the reconstruction of sparse point clouds while localizing in real-time. ORB-SLAM2 [6] adds stereo-matching and optimization algorithms, and uses stereo cameras and RGB-D cameras to achieve real-time localization and sparse reconstruction. Bescos et al. proposed DynaSLAM [7], which combines Mask R-CNN [8] and multi-view geometry to achieve culling of dynamic objects, repair the background occluded by dynamic objects, and experiment results in the KITTI Visual Odometry [9] datasets show that the algorithm has high positioning accuracy. However, due to a large number of network layers, this method is not real-time. Cui et al. proposed SDF-SLAM [10]. They developed a semantic depth filter, which makes the positioning of SLAM algorithm more accurate in a dynamic environment. They use the TUM dataset for simulations in indoor scenarios, so the performance of the algorithm in more challenging outdoor scenarios cannot be represented.

Compared with visual SLAM, Lidar-based SLAM cannot perceive the color and texture of the environment, but it has strong anti-light interference ability and can use depth information to improve positioning accuracy. The LeGo-LOAM proposed by Shan et al. [11] filters out the noise generated by the driving of unmanned vehicles by segmenting Lidar point clouds, and then extracts planar and edge features in the point clouds to reduce positioning errors, realizing the use of embedded systems to locate unmanned vehicles and build sparse maps. In scenarios with fewer point clouds on the ground, the algorithm is prone to collapse. Plane-LOAM, proposed by Ćwian et al. [12], extracts planes and line segments from Lidar point clouds to build maps, and uses factor graphs to optimize unmanned vehicle trajectories and sparse maps. The above methods have shown good performance using accurate lidar point clouds, but the sparsity of point clouds leads to a poor visibility of mapping.

### 2.2. Multi-Sensor Fusion SLAM

SLAM based on multi-sensor fusion can effectively overcome the performance limitations of a single sensor. The VINS-Mono [13] system proposed by Qin et al. combines a monocular camera and IMU to design a tightly coupled VIO (visual inertial odometry) system. The IMU pre-integration algorithm proposed in this method effectively reduces the negative impact of factors such as illumination changes and texture-missing on the localization accuracy and helps to build sparse maps. ORB-SLAM3 [14] uses maximum posterior probability estimation during IMU initialization to achieve stable operation in various scenes; the multi-map system based on scene re-identification enables the algorithm to work in low-texture environments such as tunnels and indoors. It is a robust operation, but has significant computational cost. The method designed by Ku et al. [15] uses RGB images to complete the Lidar scans, which can make up for the deficiency of the insufficient scanning angle of the Lidar scanner, to a certain extent. LIMO, proposed by Graeter et al. [16], correlates Lidar and image data through the projection of point clouds and frames to obtain depth estimates of visual features. Sparse Lidar point clouds work only in a supplemental role, so the LIMO can only build sparse maps.

Visual features often cannot be completely matched with depth maps or Lidar point clouds. The work proposed by De Silva et al. [17] uses a Gaussian process regression to interpolate missing values after computing the geometric transformation between the visual and Lidar sensors. Therefore, the Lidar is used to initialize the features detected directly in the image. However, this method performs sensor fusion in a limited space and did not perform accurately in experiments on real-time datasets, nor was it compared with other methods. The visual odometry DEMO [18] proposed by Zhang et al. combines monocular vision and depth information to classify feature points into feature points with depth information matching, feature points with depth obtained by triangulation, and feature points without depth information, then combines them for pose estimation. The experiment was conducted on the KITTI Visual Odometry datasets and the result shows that the positioning accuracy is even higher than that of some stereo methods, but the DEMO is more sensitive to the features of specific angles. DVL-SLAM [19], proposed by Shin et al., is based on the direct method, which fuses multi-line laser and monocular images, and uses pixels with depth information instead of features. They reduce the matching error between Lidar points and images through sliding window optimization. Experiment results confirm that the algorithm has high positioning accuracy and can perform the 3D reconstruction. Compared with the feature-based method, DVL-SLAM is more computationally intensive and susceptible to gradient changes.

### 2.3. Semantic SLAM

Semantic SLAM, combined with deep learning, can utilize the semantics of objects in the scene to improve performance in dynamic environments, which is helpful for more complex tasks such as recognition and obstacle avoidance [2]. Using depth maps, Fusion++ [20] achieves 3D reconstruction of indoor objects by combining Mask R-CNN and TSDF (truncated signed distance function). However, this algorithm is only suitable for indoor static environments. MaskFusion [21] uses Mask R-CNN to instances segment, and proposes a geometry-based segmentation algorithm, which in turn provides more accurate masks based on the depth and surface normal cues of the images, enabling real-time tracking in dynamic environments, reconstructing, and assigning semantic labels to the reconstructed map. DynSLAM [22], proposed by Bârsan et al., uses convolutional neural network MNC to detect and classify image backgrounds and moving objects, which can reduce the interference caused by dynamic targets to a certain extent, and then complete the 3D reconstruction of the KITTI datasets. However, it is severely limited by sensor performance and GPU memory.

Due to the unstructured, disordered, and irregular characteristics of Lidar point clouds, it is quite challenging to perform convolution and reconstruct semantic maps. RangeNet++, proposed by Milioto et al. [23], performs semantic segmentation on the 2D spherical projection of the point cloud, then uses the kNN search algorithm to post-process the segmentation results, and finally reprojects the segmentation results back to the 3D point clouds. The post-processing algorithm can reduce the misclassification caused by the discreteness, but it also makes the method more computationally expensive. SuMa++ [24], proposed by Chen et al., uses RangeNet++ to semantic-segment Lidar points, and uses a flood-filling algorithm to reduce semantic misclassification. The algorithm combines the semantic information of the point clouds to filter out the dynamic objects and adds constraints to the ICP (iterative closest point) algorithm, which improves the positioning accuracy and establishes a surfel-based 3D semantic map. However, this method has high complexity and computational cost.

To sum up, at the data level, the changing luminosity, the interference of moving objects, and the lack of texture in the environment all affect the feature-matching accuracy, resulting in a decrease in the positioning accuracy or even failure of positioning. At the task level, due to the lack of accurate depth information, it is difficult to reconstruct dense 3D maps using only monocular vision. Deep learning-based semantic SLAM algorithm can utilize Lidar point clouds or their spherical projection to realize 3D semantic reconstruction. However, the irregular Lidar point cloud leads to a large computation amount and a high complexity of deep learning, and the structural information in the scene is missing in spherical projection.

To solve the above problems, we propose a SLAM and 3D semantic reconstruction method based on the fusion of Lidar and monocular vision. The rest of the paper is structured as follows: in Section 3, we present the overall framework of the algorithm, and then propose an upsampling method for sparse point clouds and optimize localization accuracy with upsampled results. A semantic segmentation network is used to segment 2D images and correspond to upsampled Lidar point clouds to provide semantic information. Then, the mapping method is introduced, which uses fusion data to complete 3D reconstruction. In Section 4, we evaluate the accuracy of our algorithm on the KITTI Visual Odometry dataset and compare with other V-SLAM and fusion-based SLAM methods. Finally, conclusions are drawn in Section 5.

## 3. Methods

**Figure 1:**
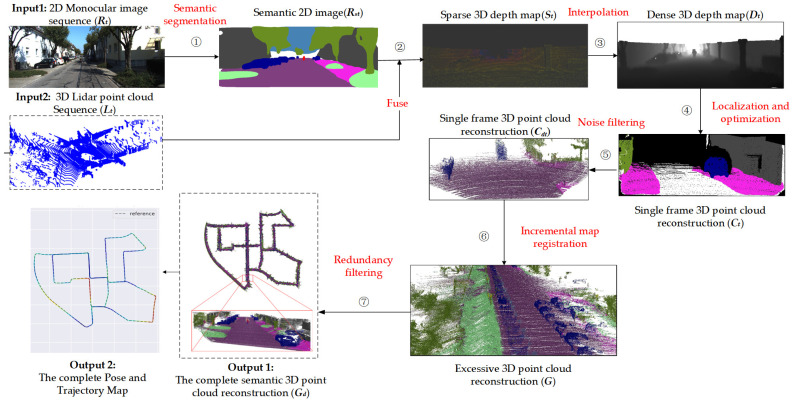
The overall framework of the proposed method. The overall framework of the proposed method, which corresponds to Algorithm 1.

Figure 1 represents the overall framework of our method, which includes the following steps. (1) Semantic segmentation. Using a BiSeNetV2-trained [25] model to semantically segment the *i*-th frame of 2D monocular image sequences of the KITTI Visual Odometry datasets, obtain the *i*-th semantic 2D image. (2) Fuse. Through the calibration parameters, project the *i*-th 3D lidar point into the corresponding *i*-th semantic 2D image to generate the *i*-th semantic sparse 3D depth map. (3) Interpolation. Using the depth interpolation algorithm to upsample the semantic sparse 3D depth map, so that the low-resolution Lidar points match the ORB feature points, output the *i*-th dense 3D points depth map. (4) Localization and optimization. Execute real-time location and mapping (ORB-SLAM2 [6]) with multithreading parallelism to generate pose/trajectory and semantic 3D point cloud reconstruction. (5) Noise filtering. Combine poses to reconstruct a single-frame 3D point cloud, and use statistical filters to eliminate outliers. (6) Incremental map registration. Using the pose information, incrementally splice 3D point clouds of each frame to obtain excessive 3D point cloud reconstruction. (7) Redundancy filtering. Use the voxel grid filter to reduce redundancy and obtain the complete semantic 3D point cloud reconstruction including the complete pose and trajectory map.

### 3.1. Data Fusion and Depth Interpolation

For existing dimensionality reduction methods, spherical projection and bird-view projection of Lidar point clouds are common methods to represent 3D point cloud data as 2D image data, and which are usually used in deep learning tasks [23]. However, these methods suffer from a critical limitation: as the point clouds are very sparse, most point clouds cannot be directly matched to 2D images to provide depth information, and the low density of point clouds also leads to missing geometric structures in fused data. In addition, these methods use a polar coordinate system to encode point clouds instead of encoding points in a Cartesian coordinate system, which may complicate front-end visual odometry.

We propose a perspective projection that projects the Lidar point cloud to the camera coordinate system, and then upsamples the fused data through a depth interpolation algorithm. We store the labels from the semantic image in the channel of the upsampled point cloud, while making the 2D image obtain additional spatial depth information. MASK-RCNN is a representative instance segmentation network [8], but its real-time performance is poor, and the long inference time seriously affects the running speed of SLAM. Therefore we use lightweight, real-time BiSeNetV2 [25] for semantic segmentation, and then use semantic images for data fusion.

Since the coordinate systems of the data collected by each device are different, we use the calibration data to project Lidar scans $L_{t}=\left\{P_{i}\in R^{3}\right\}$ into the RGB frames order by timestamp *t*, providing depth value for ORB feature points. Define Lidar point $P_{i}=\left(X,Y,Z,1\right)$ and projection point $p_{i}=\left(u,v,1\right)$ in the image. With timestamps and calibration files, $P_{i}$ is corresponded and projected to 2D image as $p_{i}$. Taking the KITTI Visual Odometry datasets as an example, $p_{i}$ is generated with Equation:

(1) $$p_{i}=\frac{1}{Z}\times P2\times R0\_\mathrm{rect}\times \mathrm{Tr}\_\mathrm{velo}\_\mathrm{to}\_\mathrm{cam}\times P_{i}$$

According to the calibration files, Tr_velo_to_cam is the projection matrix of Lidar to the grayscale camera coordinate system, R0_rect is the rotation correction matrix of the grayscale camera, P2 is the intrinsic parameter matrix of the color camera. Since the density of Lidar scans collected is much lower than the pixel density, the data generated by Lidar-vision fusion is sparse depth maps. The sparse Lidar points cannot correspond to the ORB features in the image. Therefore, we upsample the sparse depth map to improve the density of the Lidar points with Equation:

(2) $$h\left(\xi_{n^{2}-1}\right)=\frac{1}{k_{d}\left(p_{i},\xi_{n^{2}-1}\right)}\int_{-\infty }^{\infty }\int_{-\infty }^{\infty }Z\left(p_{i}\right)\omega \left(\xi_{n^{2}-1},p_{i}\right)d\xi_{n^{2}-1}$$

In dense depth maps, the projection points have a semantic information channel, which is used to associate the semantic information of corresponding pixels in the 2D images. Where $h\left(\xi_{n^{2}-1}\right)$ is the density–depth map with semantic information, $\xi_{n^{2}-1}$ is the neighbor coordinates of the square area around $p_{i}$. *Z* is the depth of $p_{i}$, ω is the calculation weight, which is the reciprocal of the spatial distance between $p_{i}$, and $\xi_{n^{2}-1}$. $k_{{}_{d}}$ is the normalization factor. Considering the time efficiency and accuracy, we set n=5 and perform depth interpolation on $5^{2}-1$ pixels around each projection point. ω and $k_{{}_{d}}$ are defined as follows:

(3) $$\omega \left(\xi_{n^{2}-1},p_{i}\right)=\frac{1}{\|\xi_{n^{2}-1}-p_{i}{\|}^{2}}$$

(4) $$k_{d}\left(p_{i}\right)=\frac{1}{\int_{-\infty }^{\infty }\int_{-\infty }^{\infty }\omega \left({\xi_{n^{2}}}_{{}_{-1}},p_{i}\right)d{\xi_{n^{2}}}_{{}_{-1}}}$$

During interpolation, we traverse the sparse fused image, compute the Euclidean distance between p and ξ, whose inverse ω is inversely proportional to the similarity between the projection points and the pixels, and assign depth values to the neighbors of the projection points.

### 3.2. Location and BA Optimization

Visual odometry (VO) is the front end of the visual SLAM algorithm, which mainly calculates the robot’s pose through images. It estimates the rough camera motion based on the information from successive images and provides a good initial value for the back end. Define vector ${\left(x,y,z,q_{0},q_{1},q_{2},q_{3}\right)}^{T}$ to describe the motion of the rigid body from initial frame $I_{0}$ to current frame $I_{k}$, where $t_{0-k}={\left(x,y,z\right)}^{T}$ describes the translation, and the quaternation ${\left(q_{0},q_{1},q_{2},q_{3}\right)}^{T}$ is converted to the rotation matrix $R_{0-k}$ to represent the roatation:

(5) $$R_{0-k}=\left[\begin{array}{ccc} 1-2q_{2}^{2}-2q_{3}^{2} & 2q_{1}q_{2}-2q_{3}q_{0} & 2q_{1}q_{3}+2q_{2}q_{0}\\ 2q_{1}q_{2}+2q_{3}q_{0} & 1-2q_{1}^{2}-2q_{3}^{2} & 2q_{2}q_{3}-2q_{1}q_{0}\\ 2q_{1}q_{3}-2q_{2}q_{0} & 2q_{2}q_{3}+2q_{1}q_{0} & 1-2q_{1}^{2}-2q_{2}^{2} \end{array}\right]$$

Then the pose T of the unmanned vehicle at time *k* is defined as a transformation matrix:

(6) $$T_{k}=\left[\begin{array}{cc} R_{0-k} & t_{0-k}\\ 0 & 1 \end{array}\right]$$

Since VO only calculates the pose of adjacent frames, the error is continuously accumulated, and finally the trajectory of the unmanned vehicle drifts. Furthermore, the rotation matrix itself is constrained (orthogonal and has a determinant of 1), which makes optimization difficult if it is used as an optimization variable. In the back end, using bundle adjustment with lie algebra and the perturbation model, we can optimize the pose of the unmanned vehicle through an unconstrained least squares problem.

**Figure 2:**
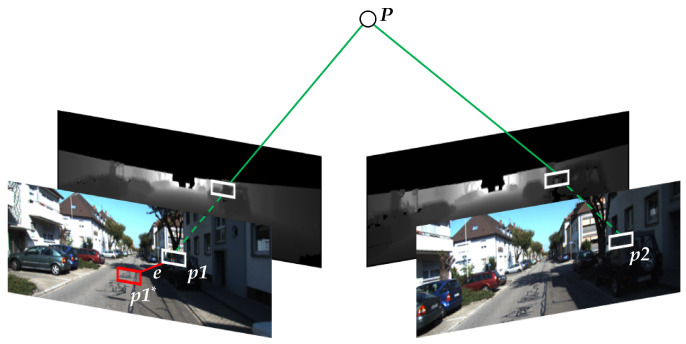
Schematic diagram of the reprojection error.

PnP (Perspective-n-Point) [26] is a method for solving the motion of 3D–2D point pairs. When we know 3D points and their projected positions, the camera pose is described by PnP. Influenced by noise, the equation of motion and the equation of observation must not be exactly equal. To solve this problem, we constructed the least squares problem, using bundle adjustment [27] to optimize the camera pose and the map point position. Figure 2 shows a schematic diagram of the reprojection error. Define the matching ORB feature points in the two frames as p1 and p2, respectively, the depth maps provide them with the true scale of depth, and P is the observation point. Under the influence of noise, P is wrongly reprojected to $p1^{*}$, and e is the reprojection error between p1 and $p1^{*}$.

For any 3D point P in the environment, the light emitted by the optical center of the camera corresponding to each view and passing through the pixels of the corresponding P in the image will intersect at P, forming a serious of beams in 3D space. Due to noise and errors during feature extraction and matching, it is almost impossible for light to converge correctly. Therefore, during the solution process, the information to be sought should be continuously adjusted so that the light can finally intersect at P [27]. The objective function to be optimized is defined as the least squares equation:

(7) $$f\left(E_{i},D_{j}\right)=argmin\sum_{\left\{i,j,k\right\}\in \chi }\rho \left({∥\underset{e}{\underset{︸}{P_{k}-h\left(E_{i},D_{j}\right)}}∥}_{}^{2}\right)$$

The quantities to be optimized are the camera pose $E_{i}$ and the position of map points $D_{j}$, where χ contains all 3D-2D projections, $P_{k}$ represents the coordinates of the image observation point, *h* is the reprojection function, e represents the cost function, and ρ is the Huber function [28] that is used to make the optimization result more robust:

(8) $$\rho =\left\{\begin{array}{c} \frac{1}{2}e^{2}\;\mathrm{if}\left|e\right|\leq \mathrm{threshold}\;\delta \\ \delta \left(\left|e\right|-\frac{1}{2}\delta \right)\;\;\;\;\mathrm{else} \end{array}\right.$$

Using the Gauss–Newton method [27] to solve the least squares problem requires providing the partial derivative of the cost function for E and D. Using the lie algebra perturbation model, the Jacobian matrices $E_{J}$ and $D_{J}$ of T and D are calculated as follows:

(9) $$\left[\begin{array}{c} X^{\prime }\\ Y^{\prime }\\ Z^{\prime } \end{array}\right]=\left[\begin{array}{cc} R & t \end{array}\right]\left[\begin{array}{c} X\\ Y\\ Z\\ 1 \end{array}\right]$$

(10) $$E_{J}=-\left[\begin{array}{cccccc} \frac{C_{x}}{Z^{\prime }} & 0 & -\frac{C_{x}X^{\prime }}{{Z^{\prime }}^{2}} & -\frac{C_{x}X^{\prime }Y^{\prime }}{{Z^{\prime }}^{2}} & C_{x}+\frac{C_{x}{X^{\prime }}^{2}}{{Z^{\prime }}^{2}} & -\frac{C_{x}Y^{\prime }}{Z^{\prime }}\\ 0 & \frac{C_{y}}{Z^{\prime }} & -\frac{C_{y}Y^{\prime }}{{Z^{\prime }}^{2}} & -F_{y}-\frac{C_{y}{Y^{\prime }}^{2}}{{Z^{\prime }}^{2}} & \frac{C_{y}x^{\prime }Y^{\prime }}{{Z^{\prime }}^{2}} & \frac{C_{x}^{\prime }}{Z^{\prime }} \end{array}\right]$$

(11) $$D_{J}=-\left[\begin{array}{ccc} \frac{C_{x}}{Z^{\prime }} & 0 & -\frac{C_{x}X^{\prime }}{{Z^{\prime }}^{2}}\\ 0 & \frac{C_{y}}{Z^{\prime }} & -\frac{C_{y}Y^{\prime }}{{Z^{\prime }}^{2}} \end{array}\right]R$$

where ${\left[X^{\prime },Y^{\prime },Z^{\prime }\right]}^{T}$ are the map point coordinates in the camera coordinate system. $C_{x}$ and $C_{y}$ are the internal parameters of the camera. The optimization problem becomes a process of gradient descent until the increments of the independent variable ΔE, the independent variable ΔD for map point position, are very small and the objective function converges to a minimum. Hence, the Gauss–Newton Equation (11) [27] can be derived to Equation (12):

(12) $$\begin{array}{c} H\left(x\right)\Delta x=-J\left(x\right)f\left(x\right) \end{array}$$

(13) $$\left[\begin{array}{cc} {E_{J}}^{T}E_{J} & {E_{J}}^{T}D_{J}\\ {D_{J}}^{T}E_{J} & {D_{J}}^{T}D_{J} \end{array}\right]\left[\begin{array}{c} \Delta E\\ \Delta D \end{array}\right]=-\left[\begin{array}{cc} E_{J} & D_{J} \end{array}\right]e$$

where the Jacobian matrix J is the first-order derivative matrix of the cost function with respect to variables, and the Hessian matrix H is the second-order matrix matrix of the cost function with respect to variables. In the optimization process, frames are first matched to obtain the initial camera pose and map point position, and then the derivative and error are calculated. The Gauss–Newton equation is solved iteratively until the objective function converges.

### 3.3. 3D Semantic Reconstruction

After VO and back-end optimization, we utilize dense depth maps with semantic information to reconstruct large-scale 3D maps for outdoor environments. Define the point $p_{i}=\left(u,v,1\right)$ in the camera coordinate system as:

(14) $$P_{c}=\left[\begin{array}{c} X=\left(u-C_{x}\right)*Z/F_{x}\\ Y=\left(v-C_{y}\right)*Z/F_{y}\\ Z=d/s\\ 1 \end{array}\right]$$

where $d\in \left[0.01,30\right]$ is the depth value, scaling factor s=1000. The internal parameters of the camera $C_{x}$, $C_{y}=707.09$, focal length $F_{x}$ = 601.89 mm, $F_{y}$ = 183.11 mm. Then the point position in the world coordinate system, that is, the coordinates in the 3D reconstructed map, is defined as:

(15) $$P_{w}=T\cdot P_{c}$$

The up-sampled semantic depth map has depth value errors, which lead to outliers in the reconstructed 3D points and destroy the geometry of the reconstruction. We use the statistical filter to traverse the reconstruction of a single frame to remove noise. The statistical filter is defined as:

(16) $$d_{\max}=\mu_{k}+\alpha \times \sigma$$

where $\mu_{k}$ is the average distance between *k* nearest points, $d_{\max}$ is the threshold, σ=0.8 represents the standard deviation of the mean distance, scale factor α=0.8. If $\mu_{k}>d_{\max}$, the point is considered to be outlier and eliminated.

After incrementally accumulating the reconstruction of the single frame, there are a lot of redundant points in the overlapping area that consume too much memory. To reduce redundancy and optimize the geometric structure of the 3D reconstruction, we use voxel grid filtering to construct several voxel grids in reconstruction, then calculate the center of gravity of the point cloud and eliminate the points around the center of gravity, which are defined as follows:

(17) $$V=\frac{1}{n}\sum_{i=1}^{n}p_{i}$$

where *V* is the center of gravity of the voxel grid, *n* is the total amount of point clouds in the voxel grid, and $p_{i}$ is the point cloud in the voxel grid. We set the voxel grid as a cube with a side length of 20 cm.

The SLAM and 3D semantic reconstruction method based on the fusion of Lidar and monocular vision is shown in Algorithm 1, which corresponds to Figure 1.
**Algorithm 1**: SLAM and 3D semantic reconstruction based on the fusion of Lidar and monocular vision.1 **input**: time stamps t, 2D monocular image sequence $R_{t}\in N^{2}$, 3D Lidar scans $L_{t}\in R^{3}$2 **output**: unmanned vehicle pose and trajectory T, complete 3D point cloud reconstruction $G_{d}$3 **begin**4 **for** t=[1,2...N]
**do**5    Semantic segments $R_{t}$ with BiseNetV2, get semantic image $R_{st}$6    Use Equation (1) to generate sparse-depth map $S_{t}$ with semantic information7    Use Equation (2)~(4) to upsampling $S_{t}$ to generate semantic dense 3D depth map $D_{t}$8    Combine $R_{t}$ and $D_{t}$ to extract ORB feature point with depth9    **if** the depth of the feature point is valid **do**10       Use Equation (5)~(13) to localizaion and potimize T11       Use Equation (14)~(15) to reconstruct semantic 3D point cloud $C_{t}$ of $D_{t}$12       Use Equation (16) to filter noise in $C_{t}$ to get $C_{dt}$13       Incremental stitching $C_{dt}$, get complete semantic 3D point cloud reconstruction G14    **end if**15 **end for**16 Use Equation (17) to filter the redundancy in G, get $G_{d}$17 **End**

## 4. Experiment and Analysis

The experimental platform used in this paper consists of CPU Intel Xeon E3-1230, GPU Nvidia GTX1080Ti, 16GB memory, equipped with Ubuntu 16.04. We use the public dataset KITTI Visual Odometry [9] and CityScapes [29] for experiments.

### 4.1. Semantic Segmentation

The CityScapes datasets use vehicle-mounted cameras to record urban environment data and have detailed semantic annotations. Therefore, we use the CityScapes datasets to train Mask R-CNN. The annotation files are saved in JSON format. The CityScapes datasets has a total of 5000 street scene images, of which 2975 images are used for training, 500 images are used for verification, and 1525 images are used for testing. The image resolution is 1024 × 2048.

The KITTI Visual Odometry datasets are collected in the city of Karlsruhe in south-western Germany, including 11 sequences from 00 to 10 with true trajectories [9], of which the sequence 01 is a highway scene, and the remaining sequences are urban or country road scenes. The RGB sequences are stored in PNG format with a resolution of 1241 × 376 and the Lidar scans are stored in BIN format with a size of about 1900 KB.

**Figure 3:**
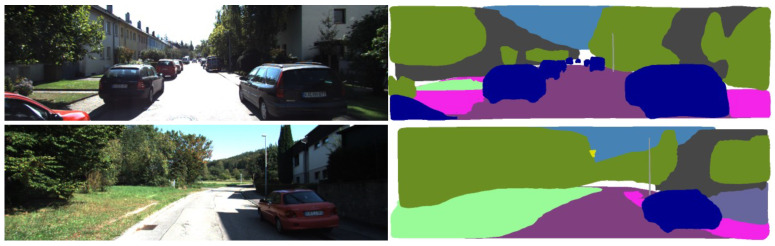
Semantic segmentation results in KITTI Visual Odometery datasets.

We trained and segmented 18 classes of objects commonly found in road scenes, using the object detection evaluation criteria as mean AP, AP50, and AP75 [30]. The trained model is applied to the KITTI Visual Odometry datasets to represent objects in the scene with different colors, and the segmentation results are shown in Figure 3. After training, the AP50 value reached 39.4, the AP75 reached 29.8, and the mean AP value was 21.8. The accuracy of 18 types of objects trained by BiSeNetV2 on the CityScape datasets is shown in Table 1. It can be seen from the table that the recognition effect of objects with larger areas is better.

### 4.2. Data Fusion and Depth Interpolation

**Figure 4:**
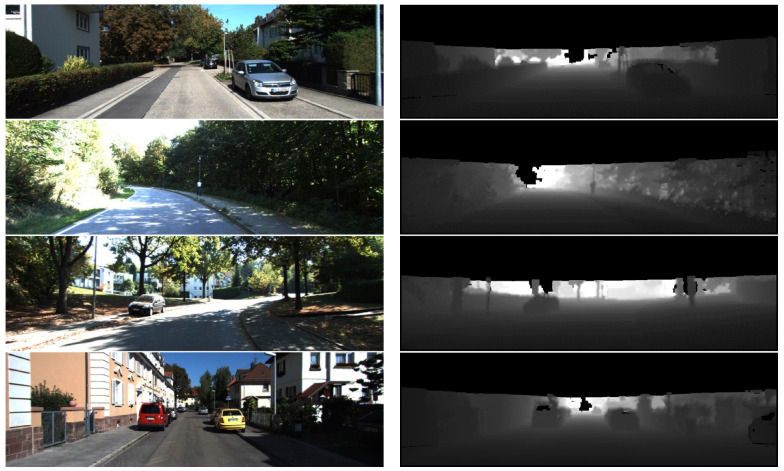
Comparison of raw images with density–depth maps.

Figure 4 shows the comparison of raw RGB images and dense-depth maps. We projected sparse Lidar scans to the semantic images and then performed deep interpolation on the fused data. In the depth maps, the black holes in the distance are the area outside the scanning range of the Lidar scanner, which is 120 m away from the location of the vehicle. Because objects that are too far away and the sky cannot reflect the emitted point clouds to the Lidar scanner, and are meanwhile limited by the vertical view field ($26.8^{\circ }$) of the Velodyne64 equipped on the KITTI data collection vehicle [9], the information in the top area of 1241 × 150 of the depth map is lost. However, as our method focuses on the traffic scenarios, the building roofs and sky in the missing regions are not needed.

Our proposed perspective projection and upsampling method converts point cloud coordinates to Cartesian coordinates instead of polar coordinates, preserving the appearance information of 2D images. Based on perspective projection and depth interpolation, we not only achieve the fusion of point clouds with semantic images but also improve the projected point density. This fusion method enables the visual odometry to directly match the monocular images and the Lidar points clouds for localization with lower complexity.

### 4.3. Positioning Accuracy Based on the Fusion of Lidar and Monocular Vision

The algorithm proposed in this paper uses RGB sequences and the density–depth maps upsampled by the depth interpolation algorithm to locate unmanned vehicles. We evaluated the positioning accuracy on KITTI Visual Odometry datasets with ATE (absolute trajectory error) [31] and translation error [32].

ATE is the direct difference between the estimated trajectory and the ground-truth, which can reflect the accuracy of the algorithm and the global consistency of the trajectory very intuitively. It should be noted that the estimated pose and ground truth are usually not in the same coordinate system, so we use the public evaluation toolkit EVO to align the two trajectories and calculate the error. For all the poses from i=1 to i=m, we calculate RMSE of ATE with the ground-truth trajectories ${\mathrm{GT}}_{i}$ and the estimated trajectories $T_{i}$ in lie algebra format, which is defined as:

(18) $$ATE=\sqrt{\frac{\sum_{i=1}^{m}trans{\left(G{T_{i}}^{-1}T_{i}\right)}^{2}}{m}}$$

The translation error standard officially proposed by KITTI is the arithmetic mean of translation errors of 100 m, 200 m…800 m multi-class equal-length trajectories, and is calculated as a percentage. The lower the trajectory error value, the higher the positioning accuracy. The translation error calculation formula is defined as:

(19) $$E_{trans}\left(F\right)=\frac{1}{\left|F\right|}\sum_{(i,j)\in F}{∥\left(GT_{j}⊖GT_{i}\right)⊖\left(T_{j}⊖T_{i}\right)∥}^{{}_{2}}$$

where F is the image sequence for recording the motion of the unmanned vehicle and GT and *T* are the are the true value and estimated value of the unmanned vehicle pose, $\left\{GT,T\right\}\in SE\left(3\right)$. $T_{j}⊖T_{i}$ is the pose transformation from frame *i* to frame *j* of the sub-segment.

**Figure 5:**
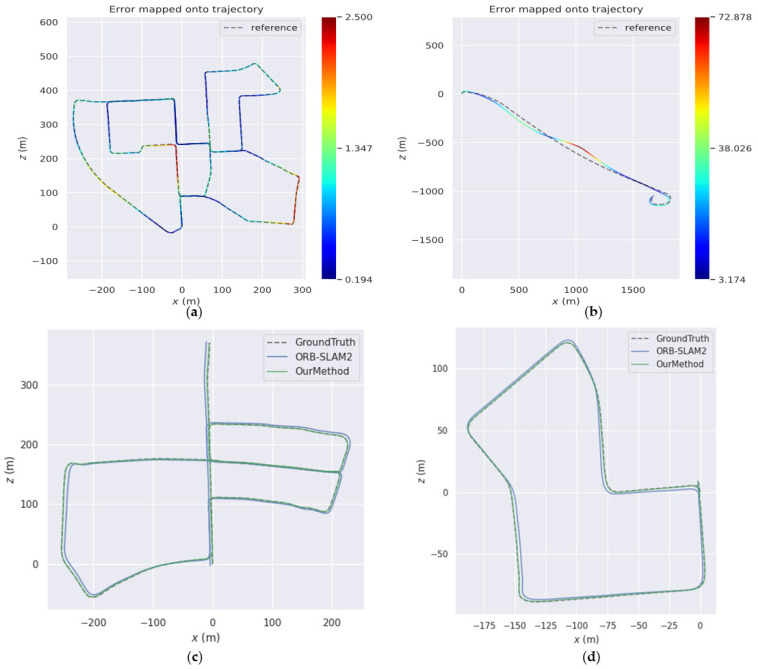
Comparison of estimated trajectories with ground truth in different sequences. (**a**) Comparison in sequence 00, (**b**) comparison in sequence 01, (**c**) comparison in sequence 05, (**d**) comparison in sequence 07.

Figure 5 shows the comparison between the estimated trajectories and the ground truth in the 00, 01, 05, and 07 sequences of the KITTI Visual Odometry datasets. In (a) and (b), the dotted lines represent the true trajectories of the unmanned vehicle, the solid lines represent the estimated trajectories, and the ATE value corresponds to the color spectrum on the right. The trajectory of each sequence is basically consistent with the ground truth, which shows that the algorithm proposed can obtain positioning results with good accuracy. In (c) and (d), we compare the estimated trajectories of our method and ORB-SLAM2; the comparisons show that our experimental results have less drift.

Table 2 compares the trajectory errors of the proposed method and monocular ORB-SLAM2 [6] and DynaSLAM [7] in the KITTI Visual Odometry datasets. In the 00 and 02~10 sequences, the average ATE of our method is 1.39 m. Compared with ORB-SLAM2 and DynaSLAM, our error is deduced by 87.51% and 87.21%, respectively. Since the sequence 01 records an empty highway scene and lacks close-range feature points that can be extracted, it is difficult to meet the feature selection criteria proposed in the paper [5], and the monocular ORB-SLAM2 and DynaSLAM algorithms cannot work. The algorithm proposed in this paper utilizes accurate depth information and does not obtain depth values through triangulation, so it can effectively locate.

**Figure 6:**
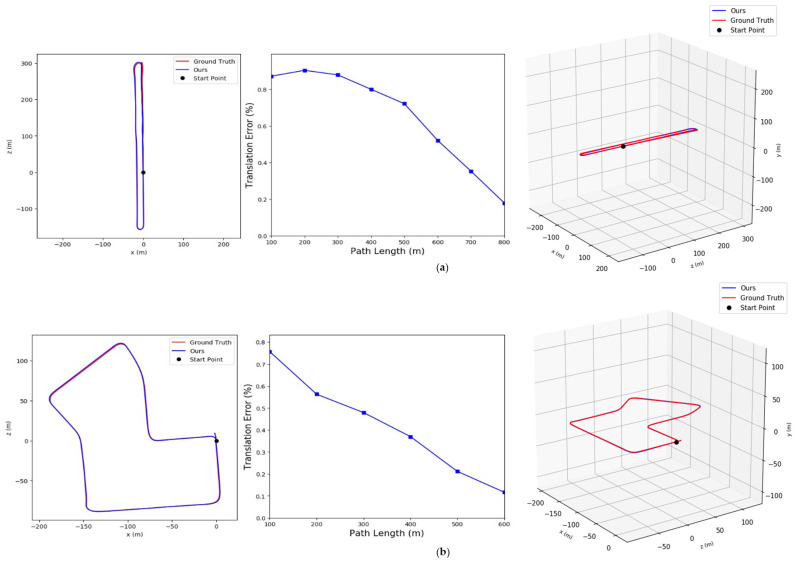
The average translation error between our method and ground-truth: (**a**) Results in sequence 06, (**b**) Results in sequence 07.

Figure 6 shows the translation errors of subsequences in the KITTI Visual Odometry datasets. The red curves are the ground truth, the blue curves are the estimation results, and the blue polylines represent the translation error of each subsequence. It can be seen that the error between our computed trajectories and the ground truth remains within a small variation and the changes in the estimated trajectories in the three dimensions are similar to ground-truth trajectories.

We compared our method with other multi-sensor fusion SLAM. In Table 3, we compare the average translation error of our method with DEMO [18] and DVL-SLAM [19] in the KITTI Visual Odometry datasets. Out of 11 sequences, we outperform DEMO in 7 sequences and DVL-SLAM in 6 sequences. The Velodyne64 Lidar scanner used by KITTI has a detection range of 120 m, which cannot provide depth information further in the 01 sequence; some feature points are discarded, resulting in a large positioning error. In the area where depth information is missing, DEMO estimates the depth of feature points by triangulation, which retains more feature points than our method; DVL-SLAM is based on the direct method, which is not affected by too few close-range feature points. Therefore, they have higher positioning accuracy in the sequence 01.

### 4.4. 3D Reconstruction

Since the KITTI Visual Odometry dataset does not provide the ground truth of objects in the 3D scene, in this section we use MeshLab [33] to measure the length of objects in the 3D reconstructed sequence 05, and use Google Earth [34] to measure the true length. Quantitative evaluation was performed using the relative error of the two measurements. We then perform 3D reconstruction of the sequence 06, and compare the reconstruction quality of DynSLAM [22] in the same sequence to conduct the qualitative evaluation. For the sake of comparison, the 3D reconstructions shown in our quantitative and qualitative assessments retain the original colors of the scenes. At the end of this section, we will show the 3D semantic reconstruction of the sequence 00, with different colors representing the classes of objects in the point clouds.

#### 4.4.1. Quantitative Evaluation of Reconstruction

**Figure 7:**
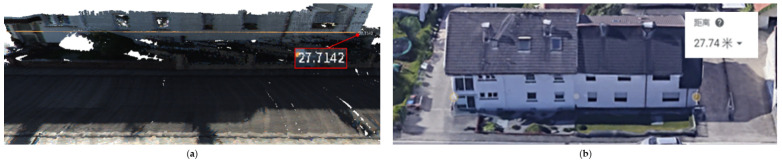
Width comparison between the reconstruction and the real building: (**a**) the length of the building measured using MeshLab is 27.7142 m. (**b**) The length of the building measured using GoogleEarth is 27.74 m.

The latitude and longitude of the 01 sequence is about $9^{\circ }03^{\prime }06.6^{\prime \prime }\;N$, $8^{\circ }23^{\prime }51.4^{\prime \prime }\;E$ [9]. In Figure 7, (a) is the schematic diagram using Meshlab to measure the 3D reconstruction length, and the measured length of the house is 27.74 m; (b) is the schematic diagram using Google Earth to measure the actual length of the house, and the length is 27.71 m. Taking the MeshLab measurement result as the observations and the measurement result of Google Earth as the true value, the reconstruction error is about 0.09%

**Figure 8:**
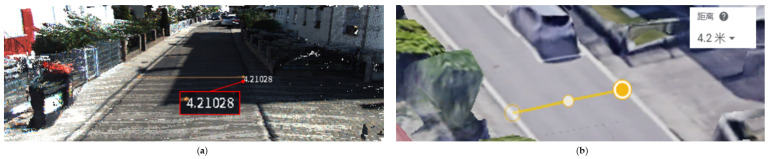
Width comparison between the reconstruction and the real building: (**a**) the length of the building measured using MeshLab is 4.21 m. (**b**) The length of the building measured using GoogleEarth is 4.20 m.

Figure 8 shows our reconstructed road width compared to Google Earth. (a) shows the width of the reconstructed road measured by MeshLab, and the width is 4.21 m; (b) shows the actual width of the road measured manually in Google Earth, and the measured width is 4.20 m. The relative error in the width of the reconstructed road is about 0.24%.

#### 4.4.2. Qualitative Evaluation of Reconstruction

**Figure 9:**
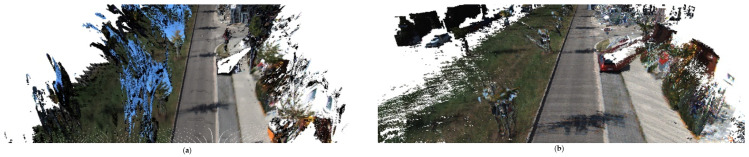
Comparison of 3D reconstruction quality of DynSLAM and our method for sequences 06: (**a**) reconstruction of DynSLAM, (**b**) reconstruction of our method.

Figure 9 shows the reconstruction quality of our method compared to DynSLAM for the same scene in the sequence 06. The total number of our reconstructed point clouds is 4.8 × 10${}^{9}$, and the total number of point clouds generated by the DynSLAM is 20 × 10${}^{9}$. Due to the small scanning angle of Lidar, we cannot perform 3D reconstruction of the full view, and the redundancy and noise are filtered, so the total amount of point clouds is relatively small and the required storage capacity is about 76% lower than that of DynSLAM.

**Figure 10:**
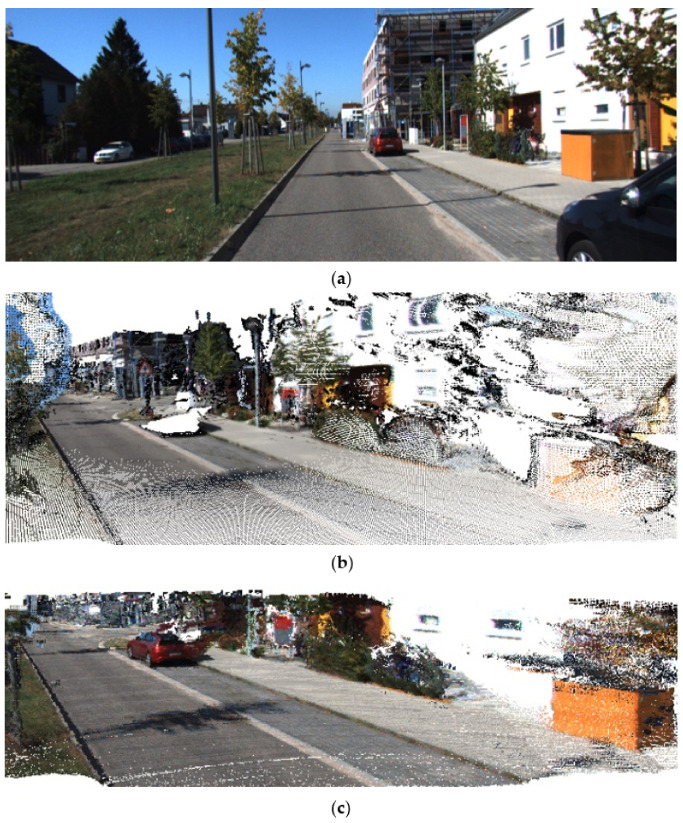
Reconstruction quality of different algorithms compared to raw image: (**a**) Original image of the sequence 06. (**b**) Reconstruction of our DynSLAM. (**c**) Reconstruction of our method.

Figure 10 shows the comparison of the reconstruction quality of the same wall between our method and the DynSLAM algorithm. (a) is the 10th picture of the sequence 06. Taking the white wall on the right part of (a) as a reference, our reconstruction quality in (c) is compared with DynSLAM in (b). It can be seen from the comparison that the wall reconstructed by DynSLAM is heavily distorted, but our method recovers the wall geometry correctly.

**Figure 11:**
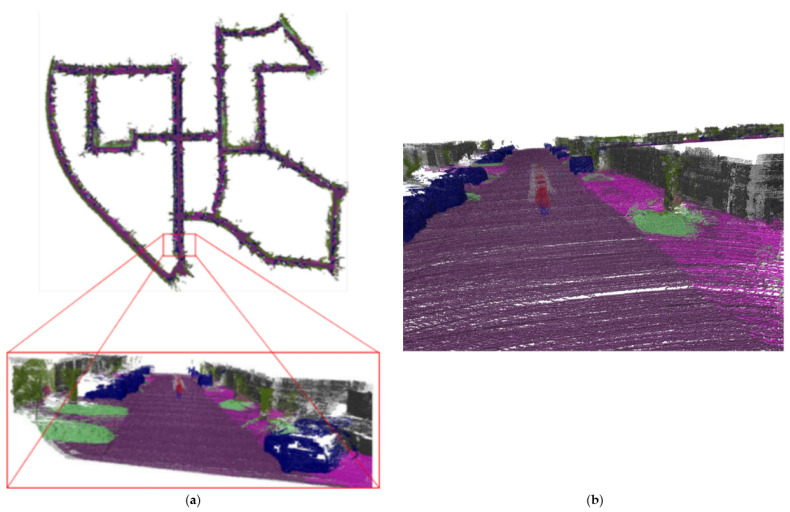
Semantic reconstruction of sequence 00 and local details: (**a**) The complete semantic reconstruction of sequence 00. (**b**) The magnified local details of semantic reconstruction.

Sequence 00 records that the dimension is 564 m × 496 m for the urban scene. Figure 11 shows the result of sequence 00 reconstruction, and the vehicles, lawns, trees, roads, and other objects on the map are represented in different colors. The semantic reconstruction maintains the geometric structure of the scene, and the unmanned vehicle uses the semantic information in the reconstructed map to help complete obstacle avoidance and navigation tasks. Daniel et al. [35] fuse semantic images from a camera with Lidar point clouds, and their focus is on off-road terrains. In contrast, our method can be applied to urban driving scenarios. Similar to our method, to maintain and automatically label large-scale 3D point cloud maps, David et al. [36] also projects the Lidar point clouds to semantically segmented 2D images, and associates the point clouds with semantic labels. However, in order to infer the underlying geometric features of sparse Lidar points, it is necessary to drive once within the experimental region, extracting small dense regions of a previously built dense map, and then semantically reconstruct it. Our method solves the sparsity problem through depth interpolation. When the unmanned vehicle is driving for the first time, the localization and semantic reconstruction can be performed simultaneously, without the need to build a map in advance. By fusing Lidar and a monocular camera, our method is capable of automatically generating representations of the world with road features in a single pass of the scene without manual labeling.

## 5. Conclusions

In this paper, we propose an SLAM and 3D semantic reconstruction method based on the fusion of Lidar and monocular vision. We designed a depth interpolation algorithm and used a semantic segmentation network BiSeNetV2 to realize the fusion of semantics images and Lidar scans. The accurate depth information provided by Lidar points is used to optimize the localization accuracy of feature-based V-SLAM. We also added a dense mapping thread, which combines the pose and semantic information to simultaneously reconstruct the 3D semantic map of the outdoor scene while locating the unmanned vehicle. Through experimental verification on the KITTI Visual Odometry dataset, we can draw the following conclusions:

(1) Based on projection and interpolation methods, we implement upsampling of sparse Lidar point clouds and fusion with high-resolution 2D images. Semantic segmentation images are used to provide semantic information. The experimental results show that the fusion data has high resolution and visibility, and can be used as an input to realize the operation and experimental verification of subsequent algorithms.
(2) Our method is compared with vision-based SLAM methods and Lidar vision-fusion SLAM methods, and the experiments were conducted on the KITTI Visual Odometry datasets. Results show that the positioning errors of our method in all 11 sequences are greatly reduced compared to ORB-SLAM2 and DynaSLAM. Compared with DEMO and DVL-SLAM, based on Lidar-vision fusion, the positioning accuracy of our method is also improved.
(3) In terms of 3D reconstructions, our reconstructed objects’ widths differ by less than 0.5% compared to the data measured in Google Earth. Compared to DynSLAM for 3D reconstruction of outdoor environments, our reconstructions are of a higher quality and require 76% less storage space. The map representation can be continuously updated over time. At the same time, the generated semantic reconstruction has labels that can be understood by humans. It can better support the practical application of autonomous driving technology in the future.

In the future, we will consider fusing more sensors to improve localization accuracy and reconstruction quality, such as by combining cameras and IMUs to build VIO systems that make algorithms work better in scenes that lack extractable feature points.

## Figures and Tables

**Table 1:** Recognition accuracy of objects in CityScapes.

Catagory	AP	AP50	AP75	Color
Sky	31.6	66.3	25.4	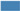
Road	81.5	96.6	95.5	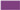
Sidewalk	21.6	42.4	19.2	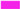
Plant	18.4	38.6	15.4	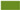
Pillar	5.2	13.6	3.0	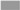
House	20.6	43.9	18.2	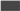
Traffic signal	15.9	29.4	15.6	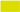
Fence	4.0	10.3	2.2	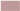
People	22.9	43.9	20.9	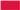
Car	39.0	61.7	41.3	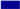
Bike	17.4	37.8	13.4	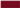
Rider	24.3	45.9	24.0	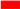
Traffic light	10.9	27.0	6.7	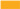
Terrain	8.6	21.5	4.8	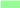
Truck	24.7	39.3	26.9	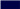
Motorcycle	12.8	30.8	7.4	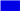
Train	16.3	36.4	6.9	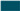
Bus	34.1	52.7	38.4	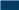

**Table 2:** Comparison of ATE (absolute trajectory error) on the KITTI Visual Odometry datasets.

Sequence	Scene	Length (m)	ORB-SLAM2 (m)	DynaSLAM (m)	Ours (m)
00	Urban	3714	5.33	7.55	1.10
01	Highway	4268	Fail	Fail	32.52
02	Country + Urban	5075	21.28	26.29	2.76
03	Country	563	1.51	1.81	0.40
04	Country	397	1.62	0.97	0.32
05	Urban	2223	4.85	4.60	0.65
06	Urban	1239	12.34	14.74	0.88
07	Urban	695	2.26	2.36	0.38
08	Urban	3225	46.68	40.28	5.39
09	Country + Urban	1717	6.62	3.32	1.11
10	Country + Urban	919	8.80	6.78	0.86
	Mean		11.13	10.87	1.39

**Table 3:** The average translation error of different algorithms on the KITTI Visual Odometry datasets.

Sequence	Scene	DEMO (%)	DVL-SLAM (%)	Ours (%)
00	Urban	1.05	0.93	0.86
01	Highway	1.87	1.47	8.10
02	Country + Urban	0.93	1.11	1.07
03	Country	0.99	0.92	1.47
04	Country	1.23	0.67	0.68
05	Urban	1.04	0.82	0.62
06	Urban	0.96	0.92	0.72
07	Urban	1.16	1.26	0.52
08	Urban	1.24	1.32	1.30
09	Country + Urban	1.17	0.66	0.88
10	Country + Urban	1.14	0.70	0.95

## Data Availability

The datasets used in the paper are the public KITTI Visual Odometry and CityScapes. They can be downloaded at: https://www.cvlibs.net/datasets/kitti/eval_odometry.php and https://www.cityscapes-dataset.com/ (accessed on 19 March 2022).
